# M19 Modulates Skeletal Muscle Differentiation and Insulin Secretion in Pancreatic β-Cells through Modulation of Respiratory Chain Activity

**DOI:** 10.1371/journal.pone.0031815

**Published:** 2012-02-20

**Authors:** Linda Cambier, Patrice Rassam, Béatrice Chabi, Karima Mezghenna, René Gross, Eric Eveno, Charles Auffray, Chantal Wrutniak-Cabello, Anne-Dominique Lajoix, Pascal Pomiès

**Affiliations:** 1 CNRS UMR5237, Centre de Recherche en Biochimie Macromoléculaire, Montpellier, France; 2 INRA UMR866, Dynamique Musculaire et Métabolisme, Montpellier, France; 3 CNRS UMR5232, Centre for Pharmacology and Innovation in Diabetes, Montpellier, France; 4 Université Montpellier 1, Montpellier, France; 5 Université Montpellier 2, Montpellier, France; 6 Genexpress, Functional Genomics and Systems Biology for Health, CNRS Institute of Biological Sciences, Villejuif, France; 7 INSERM U1046, Physiologie et Médecine Expérimentale du Coeur et des Muscles, Montpellier, France; Inserm, France

## Abstract

Mitochondrial dysfunction due to nuclear or mitochondrial DNA alterations contributes to multiple diseases such as metabolic myopathies, neurodegenerative disorders, diabetes and cancer. Nevertheless, to date, only half of the estimated 1,500 mitochondrial proteins has been identified, and the function of most of these proteins remains to be determined. Here, we characterize the function of M19, a novel mitochondrial nucleoid protein, in muscle and pancreatic β-cells. We have identified a 13-long amino acid sequence located at the N-terminus of M19 that targets the protein to mitochondria. Furthermore, using RNA interference and over-expression strategies, we demonstrate that M19 modulates mitochondrial oxygen consumption and ATP production, and could therefore regulate the respiratory chain activity. In an effort to determine whether M19 could play a role in the regulation of various cell activities, we show that this nucleoid protein, probably through its modulation of mitochondrial ATP production, acts on late muscle differentiation in myogenic C2C12 cells, and plays a permissive role on insulin secretion under basal glucose conditions in INS-1 pancreatic β-cells. Our results are therefore establishing a functional link between a mitochondrial nucleoid protein and the modulation of respiratory chain activities leading to the regulation of major cellular processes such as myogenesis and insulin secretion.

## Introduction

Mitochondria are cellular organelles involved in various critical cell functions including ATP production, apoptosis, calcium homeostasis and production of oxygen species. Mitochondria contain their own DNA that is found in association with proteins in organized structures called mitochondrial nucleoids. These structures, that are thought to associate with the mitochondrial inner membrane, have been shown to be essential for the protection, maintenance and propagation of mitochondrial DNA (mtDNA). The 37 genes present in the mtDNA encode mitochondrial proteins, the large and small rRNA, and 22 tRNA. In humans, while only 13 mitochondrial genes encode mitochondrial proteins, all part of the respiratory chain, it is estimated that more than 1,500 mitochondrial proteins are encoded by nuclear DNA (nDNA) [1], while only half of them has been identified [2]. These nuclear gene-encoded proteins are translated in the cytosol and therefore need to be transported across one or both mitochondrial membranes using specific targeting sequences that direct them to the different mitochondrial subcompartments [3], [4].

Numerous studies have shown that mitochondria are implicated in the regulation of cell differentiation. Indeed, it has been shown that mitochondrial protein synthesis is essential for erythroleukemia differentiation [5], that mitochondrial translation is necessary for neuroblastoma differentiation [6], and that changes in mitochondrial activity are closely associated with differentiation of osteoblasts [7]. In avian myoblasts, alteration in mitochondrial activity occurs before terminal differentiation [8]. Moreover, inhibition of mitochondrial protein synthesis by tetracycline in murine myoblasts leads to the impairment of muscle differentiation accompanied by the down-regulation of some muscle-specific genes such as muscle creatine kinase and troponin I, but does not affect myogenin and MyoD expression levels [9]. More recently, it has been demonstrated that inhibition of mitochondrial translation by chloramphenicol in avian myoblasts results in a reversible inhibition of muscle differentiation associated with a marked decrease of myogenin expression but not of the two other muscle-specific transcription factors, MyoD and Myf5 [10].

Studies have also demonstrated the importance of mitochondria in the control of insulin secretion by the pancreatic β-cell. Indeed, use of drugs affecting the respiratory chain, mutations in and depletion of the mitochondrial genome have highlighted the critical role of mitochondrial activities on glucose-stimulated insulin secretion. In this cell type, mitochondrial ATP production appears to be a key factor linking intracellular glucose metabolism and exocytosis of insulin granules, showing the importance of mitochondria in pancreatic β-cells [11]. Moreover, mitochondrial defects, including increased production of reactive oxygen species, elevated uncoupling protein 2 activity and mitochondrial DNA mutations, may participate in the impairment of glucose-induced insulin secretion of pancreatic β-cells observed in type 2 diabetes [12].

In a recent study, a novel mitochondrial nucleoid protein, M19, has been identified in HeLa cells [13]. In order to specify the cellular role of this newly described protein, we have characterized a 13-long amino acid sequence located at the N-terminus of the protein that targets the protein to mitochondria. Furthermore, using RNA interference and over-expression strategies, we have shown that mitochondrial respiratory chain activities, such as oxygen consumption and ATP production, are regulated by M19 expression levels. Finally, we have demonstrated that M19, through its modulation of the respiratory chain activity, is a positive regulator of late skeletal muscle differentiation and insulin secretion by pancreatic β-cells. Altogether, these data show the key role of a novel mitochondrial nucleoid protein in physiological processes such as mitochondrial ATP production, muscle differentiation and insulin secretion.

## Results

### Expression and cellular localization of M19 in muscle cells

Recently, a novel mitochondrial protein, called M19, preferentially expressed in brain, kidney, heart and skeletal muscle, has been identified [13]. It has been shown that human M19 is associated with mitochondrial nucleoids in HeLa cells and that it is likely present in the peripheral region of nucleoids where it could be involved in mtDNA translation and/or assembly of respiratory complexes [13].

**Figure 1:**
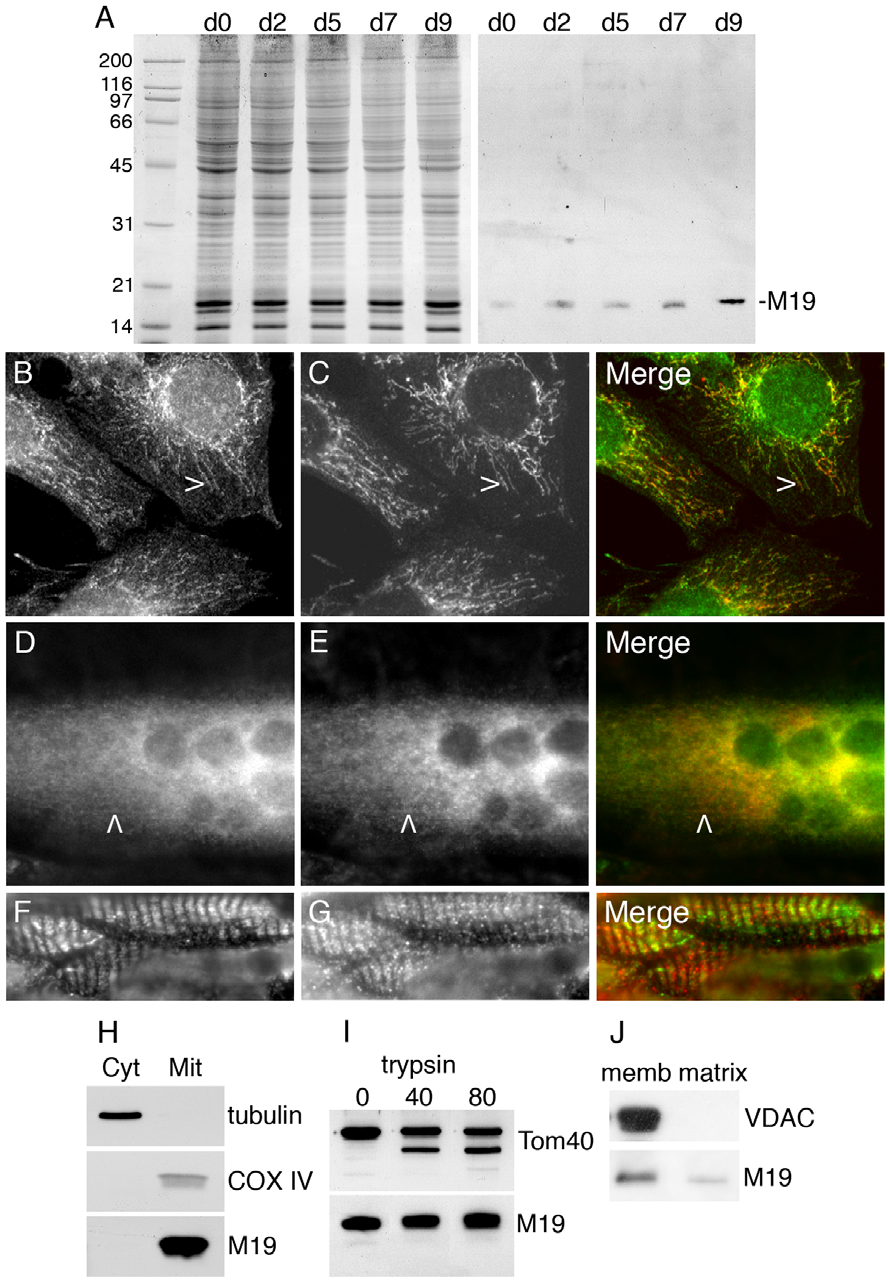
Expression and localization of M19 in muscle cells. (**A**) Coomassie-blue stained gel and Western blot analysis of M19 in extracts from C2C12 cells grown in proliferation medium (d0) or placed in differentiation-promoting conditions for 2 to 9 days (d2 to d9). M19 is detected by the rabbit polyclonal P70612 antibody. (**B**, **C**) C2C12 myoblasts were grown in proliferation medium or (**D**, **E**) were placed in differentiation medium for 6 days, and then were double-labeled with the specific P70612 antibody (**B**, **D; green**) and an anti-cytochrome c antibody (**C**, **E; red**). There is a co-localization between the 2 detected proteins in C2C12 myoblasts (**B**, **C, merge**) and myotubes (**D**, **E, merge**) as indicated by arrowheads. (**F**, **G**) Double-label indirect immunofluorescence of mouse *Tibialis anterior* sections showing M19 (**F; green**) and cytochrome c (**G; red**). (**H**) After C2C12 cell fractionation, proteins from the cytosolic and the mitochondria fractions were separated by SDS-PAGE. Tubulin, COX IV and M19 are detected by Western immunobloting. (**I**) Purified mitochondria are subjected to limited degradation using increasing concentration of trypsin, from 0 to 80 µg/ml. The mitochondria are then lysed in Laemmli buffer. Tom40 and M19 are detected by Western immunobloting. (**J**) Purified mitochondria are disrupted with freeze/thaw cycles, followed by Na_2_CO_3_ precipitation. After centrifugation, the membrane fraction (memb) and the matrix/intermembrane space fraction (matrix) are analyzed by Western immunobloting using a VDAC antibody and the P70612 antibody.

In order to gain insight into the function of M19 in muscle cells, we have generated a rabbit polyclonal antibody, called P70612, from the immunization of a rabbit with the bacterially expressed human M19. We used the murine C2C12 cell line to determine the expression level of the protein during skeletal muscle differentiation. The purified polyclonal antibody P70612 recognized predominantly a single band at about 18 kDa, in accordance with a predicted molecular weight of 16.3 kDa for the mouse protein (Figure 1A). Mouse M19 is detected in proliferating myoblasts and its expression increases progressively upon induction of differentiation (Figure 1A), highlighting its potential role during differentiation/regeneration processes and in differentiated skeletal muscle. We next examined the cellular localization of mouse M19 in C2C12 cells, using the specific P70612 antibody. As seen in figure 1B-E, M19 colocalizes with cytochrome c in the mitochondria of C2C12 myoblasts and myotubes. In myoblasts, the studied protein is detected in short filaments and dots (Figure 1B), while in myotubes, the staining is more diffuse even though some staining is detected in faint filaments (Figure 1D). Longitudinal sections of mouse *Tibialis anterior* muscles were processed with the P70612 antibody. Examination of these sections showed regular transversely oriented rows of staining, similar to what is observed for cytochrome c staining (Figure 1F, G).

To confirm the mitochondrial localization of mouse M19 in C2C12 cells, we performed cell fractionation experiments using C2C12 myoblasts. Immunoblot analyses of isolated mitochondria extracts and cytosolic fractions of C2C12 cells show that tubulin is found in the cytosolic fraction while, as expected, the mitochondrial extract is greatly enriched in COX IV, a mitochondrial enzyme (Figure 1H). In accordance with the mitochondrial localization of mouse M19 observed by fluorescence microscopy, the studied protein is detected in the mitochondrial extract. Furthermore, we subjected purified mitochondria from C2C12 myoblasts to limited trypsinization. This treatment allowed the degradation of cytosolic proteins interacting with the mitochondrial outer membrane or intrinsic proteins of the outer membrane. As a marker protein of the outer mitochondrial membrane, we used the import receptor, Tom40. When intact mitochondria were treated with increasing concentration of trypsin, Tom40 was subjected to degradation while M19 was protected, suggesting that the studied protein is not loosely bound to the outer membrane or an intrinsic protein of the outer membrane (Figure 1I). To further determine the intra-mitochondrial localization of M19, we disrupted purified mitochondria with freeze/thaw cycles and then subjected the homogenate to Na_2_CO_3_ precipitation followed by centrifugation. This technique allowed us to separate the mitochondrial membrane fraction from the matrix/intermembrane space fraction. Immunoblot analyses showed that the mitochondrial marker VDAC is found, as expected, in the membrane fraction. Interestingly, M19 is mostly detected in the membrane fraction even though some protein is also present in the matrix/intermembrane space fraction (Figure 1J). These digestion and fractionation experiments strongly indicate that mouse M19 resides within mitochondria of C2C12 cells, likely at the interface between the matrix and the inner membrane, in accordance with the mitochondrial localization of M19 previously described in HeLa cells [13].

### M19 contains a functional mitochondrial targeting signal

**Figure 2:**
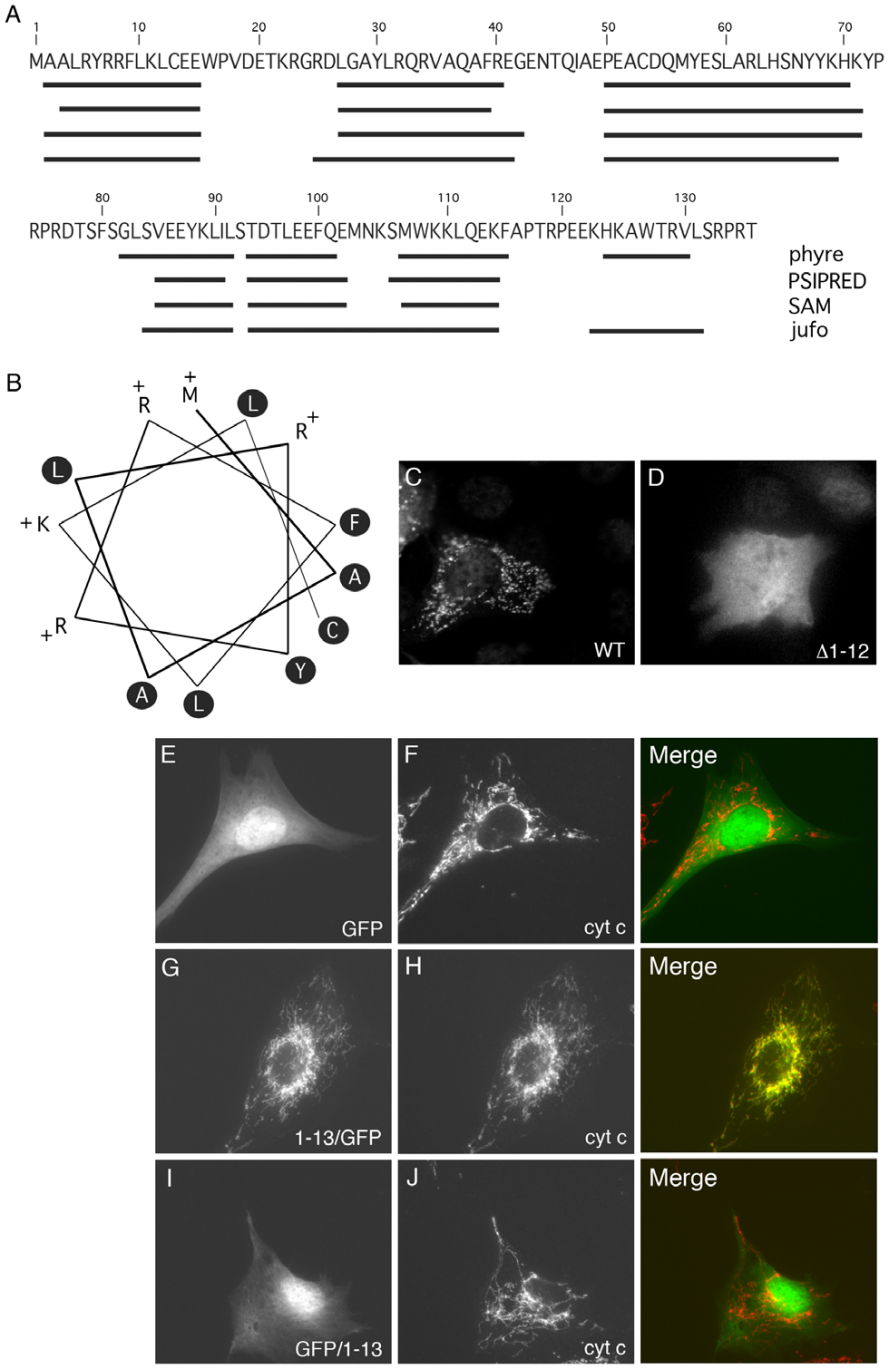
Identification of a mitochondrial targeting signal. (**A**) Prediction of the secondary structure of mouse M19 (*Mus musculus* NM026063) using 4 different algorithms: phyre, PSIPRED, SAM and jufo. The predicted α-helices are indicated by black lines along the amino-acid sequence. (**B**) Helical wheel presentation of the N-terminal α-helix of mouse M19, from amino acid 1 to 13. Hydrophobic residues are indicated in black circles while the positively charged amino acids are mentioned with a “+”. The first methionine (amino acid 1), at the top of the figure, is considered as a positively charged residue. (**C**, **D**) C2C12 myoblasts were transfected with the pQETriSystem vector encoding histidine-tagged M19 (**C**) or a histidine-tagged M19 mutant lacking amino acids 1 to 12 (**D**). Indirect immunofluorescence was performed using an anti-histidine antibody. (**E**–**J**) C2C12 myoblasts were transfected with the pEGFP-N1 vector encoding GFP alone (**E**, **F**), the pEGFP-N1 vector encoding the N-terminal M19 α-helix fused to the N-terminal end of GFP (**G**, **H**), and the PEGFP-C3 vector encoding the N-terminal M19 α-helix coupled to the C-terminal end of GFP (**I**, **J**). Fluorescence microscopy allows the direct detection of GFP constructs (**E**, **G**, **I; green**) and the indirect detection of cytochrome c using an anti-cytochrome c antibody (**F**, **H**, **J; red**).

Despite its mitochondrial nucleoid association, Sumitani *et al*. did not describe any mitochondrial localization signal within human M19 [13]. Nevertheless, the mitochondrial localization of M19, the prediction of a potential cleavage site for a mitochondrial targeting sequence between amino acid 9 and 10 (WoLF PSORT software; http://wolfpsort.org/; data not shown), and the fact that the protein is encoded in the nucleus led us to search for the presence of a potential mitochondrial localization signal within the protein. For most of the proteins targeted to the mitochondrial matrix or the inner membrane, this signal is an amphipathic α-helix localized at the N-terminus of the protein. We therefore used four different algorithms to predict the secondary structure of M19 from the primary sequence of the mouse protein. The programs predicted the presence of 6 to 7 α-helices within the protein, with one localized at the N-terminus from amino acids 2 to 15 (Figure 2A). In order to determine whether this predicted N-terminal α-helix is an amphipathic helix, an helical wheel presentation of the 13 N-terminal amino acids of mouse M19 was generated as shown in Figure 2B. Interestingly, all positively charged residues lie on one face of the putative helix while the opposite face contains only hydrophobic residues. Furthermore, helical wheel projections of the other predicted α-helices of the protein showed no amphipathic feature (data not shown). This N-terminal helical conformation corresponds therefore to an amphipathic helical structure characteristic of mitochondrial targeting signals.

To test whether the putative N-terminal amphipathic α-helix of M19 is a functional mitochondrial targeting signal, we generated a N-terminal truncation, Δ1–12, which deletes amino acids 1 to 12 corresponding to a major part of the helix. Wild-type M19 and the Δ1–12 mutant, coupled to a C-terminal histidine tag, were expressed into C2C12 myoblasts. As can be seen in Figure 2C, the wild-type protein is targeted to the mitochondria, while the Δ1–12 truncation completely abolished the mitochondrial localization of the protein (Figure 2D). A similar result was obtained using a shorter truncation deleting amino acids 7 to 12 of the protein (data not shown). A more conclusive demonstration of the ability of the N-terminal amphipathic α-helix to direct mitochondrial localization was observed by fusing the amino acids 1 to 13 to the N-terminus of GFP. While GFP is localized in the cytoplasm and the nucleus of C2C12 myoblasts (Figure 2E), the 13 N-terminal amino acids of M19 clearly directed GFP to mitochondria (Figure 2G). Interestingly, when this amphipathic α-helix was fused to the C-terminus of GFP, a diffuse cytoplasmic/nuclear staining was observed (Figure 2I). Together, these data demonstrated that the N-terminal amphipathic α-helix of M19 is necessary and sufficient to direct the protein to mitochondria.

### M19 is a positive regulator of mitochondrial oxygen consumption and ATP production

**Figure 3:**
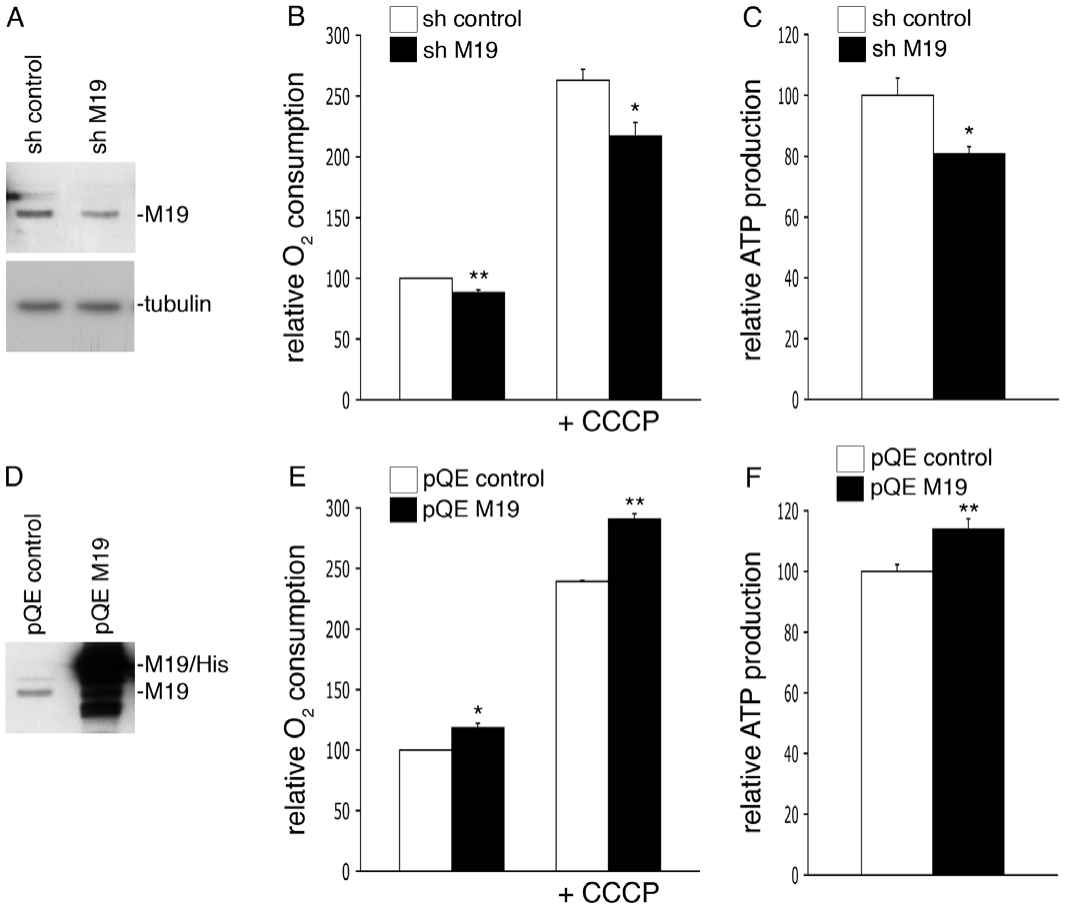
M19 expression levels regulate oxygen consumption and ATP production in C2C12 cells. (**A**–**C**) C2C12 myoblasts were transfected with a control pHYPER vector (sh control) or the pHYPER vector encoding a M19-specific shRNA (sh M19). Transfected cells were grown in proliferation medium for 2 days and were then lysed in Laemmli buffer. (**A**) Cell extracts were processed for immunoblot analysis with the P70612 antibody and an anti-tubulin antibody for loading control. (**B**) Oxygen consumption was evaluated for control cells (sh control) and for M19-specific shRNA cells (sh M19), in untreated cells or in cells treated with the ionophore CCCP. (**C**) ATP production was determined in control cells (sh control) and in M19-specific shRNA cells (sh M19). (**D**–**F**) C2C12 myoblasts were transfected with the empty pQETriSystem vector (pQE control) or the pQETriSystem vector encoding mouse M19 (pQE M19). (**D**) Cell extracts were resolved by SDS-PAGE. Endogenous M19 and exogenous histidine-tagged M19 were detected by Western immunobloting using the P70612 antibody. (**E**) Oxygen consumption was evaluated for control cells (pQE control) and for M19-overexpressing cells (pQE M19), in untreated or in CCCP-treated myoblasts. (**F**) ATP production was determined in control cells (pQE control) and in M19-overexpressing cells (pQE M19). Results are the mean ± SEM of two (**E**), three (**B**), or five (**C**, **F**) independent experiments. (*) and (**) indicate statistical significance at p<0.05 and p<0.01, respectively, according to the unpaired Student's *t* test.

M19 has been shown to be associated with mtDNA and, more precisely, it is supposed to be part of the peripheral region of mitochondrial nucleoids [13]. It has been recently suggested that mitochondrial transcription occurs in their central core while mitochondrial translation and assembly of respiratory complexes occur in the peripheral region [14]. Therefore, nucleoids appear as mitochondrial key structures essential for the proper activity of the respiratory chain. So, in order to define the cellular function of M19 in C2C12 muscle cells, we decided to determine the oxygen consumption and the ATP production levels of C2C12 cells lacking M19. To this end, we transfected C2C12 myoblasts with a shRNA vector specific for mouse M19 or an empty shRNA vector as a control. After 2 days in proliferation medium, M19 was reduced by about 25% in M19 shRNA cells (Figure 3A). In these cells, basal oxygen consumption was significantly altered by 12%, compared to control shRNA cells (Figure 3B). Addition of the mitochondria-specific ionophore CCCP, which alters mitochondrial potential, induced an oxygen consumption increase in control cells. Nevertheless, in presence of CCCP, oxygen consumption was significantly reduced in cells lacking M19 compared to control cells (Figure 3B). Concomitantly to this reduced oxygen consumption determined in M19 shRNA cells, a 19%-decrease of cellular ATP was observed in these cells (Figure 3C). In parallel, we also analyzed the effect of an overexpression of M19 on oxygen consumption and ATP production. We transfected C2C12 myoblasts with the pQETriSystem vector alone or the pQETriSystem vector encoding mouse M19 coupled to a 8-histidine tag (Figure 3D). Compared to control cells, C2C12 cells overexpressing M19 showed a significant 18%-increase in basal oxygen consumption, while in presence of CCCP this increase was of 22% (Figure 3E). Furthermore, we observed a 14%-ATP production increase in C2C12 myoblasts overexpressing M19 (Figure 3F). Altogether, these results suggest that M19 regulates oxidative phosphorylation in C2C12 cells.

**Figure 4:**
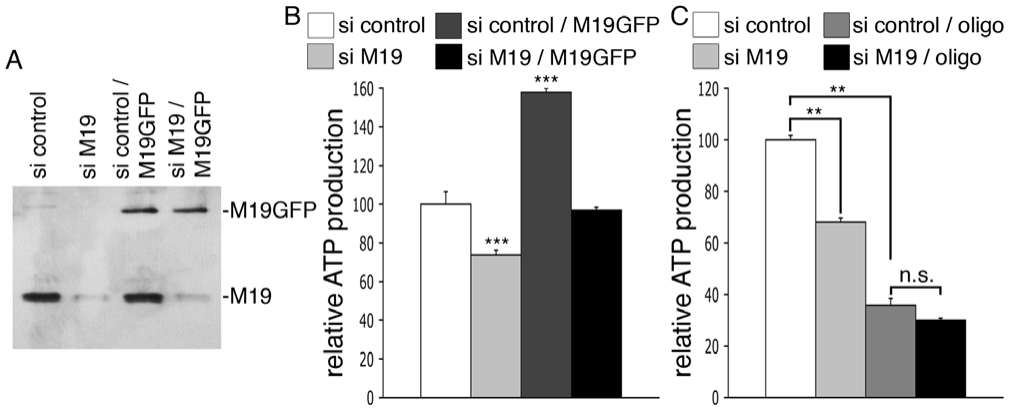
M19 expression levels regulate mitochondrial ATP production in HeLa cells. (**A**, **B**) HeLa cells were transfected with a control siRNA (si control), a human M19-specific siRNA (si M19), a control siRNA associated with the pEGFP-N1 vector encoding mouse M19 (si control/M19GFP), or a human M19-specific siRNA associated with the pEGFP-N1 vector encoding mouse M19 (si M19/M19GFP). (**A**) Expression levels of the endogenous human M19 and the mouse GFP-coupled M19 are detected by Western immunobloting using the P70612 antibody. (**B**) ATP production is presented for these transfected-HeLa cells. Results are the mean ± SEM of five independent experiments. (***) indicates statistical significance at p<0.001, according to the unpaired Student's *t* test. (**C**) HeLa cells were transfected with a control siRNA (si control) or a human M19-specific siRNA (si M19). ATP production was then determined in untreated or in oligomycin-treated cells (oligo). Results are the mean ± SEM of four independent experiments. (**) indicates statistical significance at p<0.01, and (n.s.) means statistically non significant, according to the Tukey HSD test used after performing a one-way analysis of variance.

In order to confirm these results in another cell type, we used the human HeLa cell line. M19 is detected as a single band of about 17 kDa in HeLa cell extracts using the P70612 antibody, and is localized within the mitochondria of HeLa cells as detected by indirect immunofluorescence (data not shown). HeLa cells were transfected with a siRNA specific for human M19 or with a control siRNA. The cells expressing the specific siRNA displayed a loss of M19 expression relative to control cells (Figure 4A; si control vs. si M19), accompanied with a 26%-decrease of total ATP production (Figure 4B; si control vs. si M19). Moreover, when GFP-coupled mouse M19 was overexpressed in HeLa cells, a 58%-raise of ATP production was observed (Figure 4A, B; si control vs. si control/M19GFP), showing that mouse M19 is functional in human HeLa cells. To assess the specificity of our RNA interference strategy, human HeLa cells were double transfected with the siRNA specific for human M19 and the pEGFP vector encoding mouse M19, knowing that the specific siRNA for human M19 is not able to alter mouse M19 expression (data not shown). As can be seen, overexpression of mouse M19 in M19-deficient HeLa cells (Figure 4A; si M19/M19GFP) restores ATP production in these cells (Figure 4B; si M19 vs. si M19/M19GFP). These data thus demonstrate the specificity of our RNA interference strategy and confirm that M19 plays a fundamental role in cellular ATP production.

Knowing that mitochondria are responsible for the production of the majority of cellular ATP, we have investigated whether M19 modulates cytosolic ATP or mitochondrial ATP, using oligomycin, a powerful inhibitor of mitochondrial ATP synthase. Treatment of control HeLa cells with oligomycin induces a drop in cellular ATP production corresponding to the loss of mitochondrial ATP, the remaining ATP being cytosolic ATP (Figure 4C; si control vs. si control/oligo). Interestingly, the use of a specific siRNA directed against M19 does not further reduce ATP production in oligomycin-treated cells (Figure 4C; si control/oligo vs. si M19/oligo), indicating that M19 inhibition does not affect cytosolic ATP production. Therefore, these experiments show that the mitochondrial nucleoid protein, M19, is a positive regulator of mitochondrial ATP production.

### M19 plays a role on late skeletal muscle differentiation

**Figure 5:**
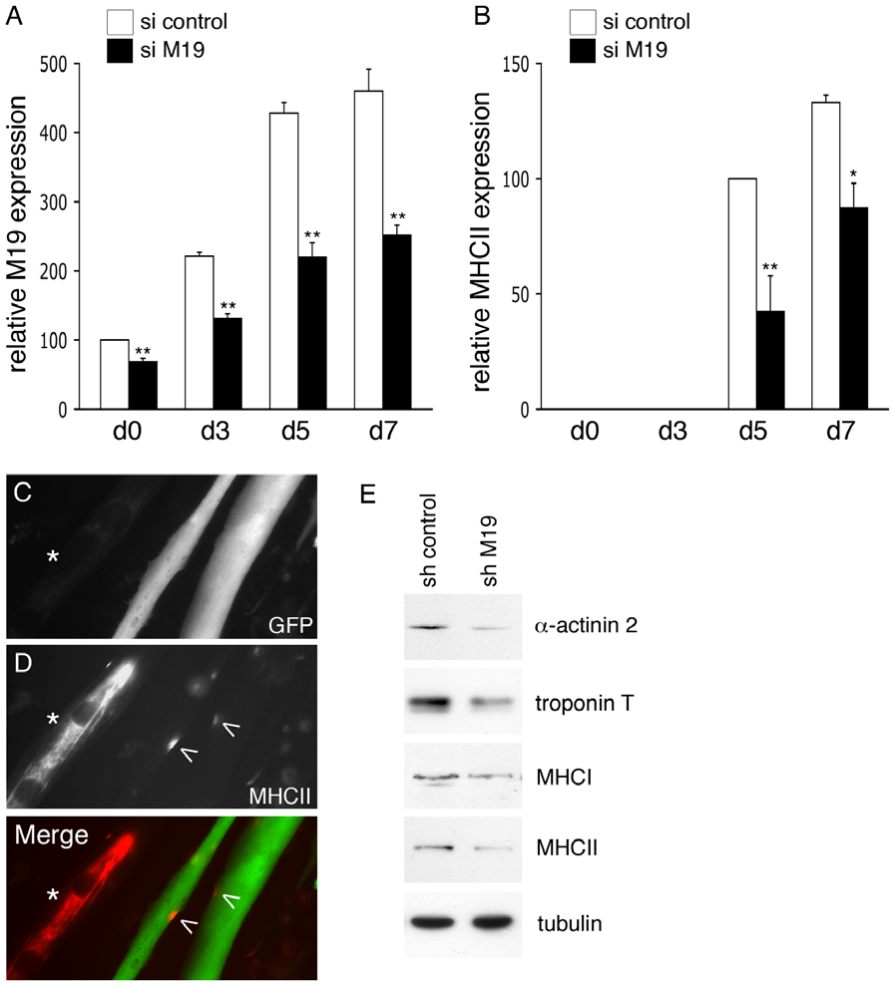
Expression of late muscle differentiation markers is affected in differentiated M19-deficient C2C12 cells. (**A**, **B**) C2C12 myoblasts were transfected with a control siRNA (si control) or a M19-specific siRNA (si M19) and were then placed in differentiation medium for 7 days. Protein extracts from transfected cells grown in proliferation medium (d0) or in differentiation-promoting conditions for 3, 5 and 7 days (d3, d5, d7) were analyzed by Western immunobloting using the M19-specific P70612 antibody (**A**) and a MHCII antibody (**B**). Densitometry analysis of the detected bands is presented as the relative expression of M19 (**A**) and MHCII (**B**) normalized to tubulin. Results are the mean ± SEM of three independent experiments. (*) and (**) indicate statistical significance at p<0.05 and at p<0.01. In a similar experiment, C2C12 myoblasts were transfected with the M19-specific shRNA vector allowing the expression of the specific shRNA with GFP. Cells were placed in differentiation medium for 7 days. Fluorescence microscopy allows the direct visualization of GFP-labeled cells expressing the M19-specific shRNA (**C, merge; green**) and the detection of MHCII using an anti-MHCII antibody (**D, merge; red**). (**E**) Protein extracts from control shRNA-transfected C2C12 cells (sh control) and M19-specific shRNA-transfected C2C12 cells (sh M19) grown in differentiation-promoting conditions for 7 days were analyzed by Western immunoblotting. The expression of the late muscle differentiation markers α-actinin 2, troponin T, MHCI and MHCII is shown, as well as the control protein tubulin.

It has been previously shown that impairment of mitochondrial function by various drug treatments leads to the inhibition of muscle differentiation [9], [10], [15]. Because mitochondrial ATP production is impaired in C2C12 myoblasts lacking M19, we decided to determine whether M19 expression levels could regulate muscle differentiation. Therefore, a possible inhibition of muscle differentiation was studied in M19-deficient muscle cells. C2C12 myoblasts transfected with a M19-specific siRNA were placed in differentiation-promoting conditions for 7 days, and M19 expression levels were detected by Western immunoblotting. As can be seen in Figure 5A, during the entire differentiation process, M19 expression is severely reduced in specific siRNA cells compared to control cells. As a marker of late differentiation, the expression levels of MHCII were determined in these M19-deficient cells. Concomitantly to the reduction of M19, a loss of MHCII expression was observed during the differentiation process (Figure 5B), suggesting that late myogenesis is affected in cells lacking M19. In accordance with this result, a loss of expression of the late differentiation marker troponin T was also observed in M19-deficient C2C12 cells placed in differentiation conditions for 5 to 7 days (data not shown). However, neither the expression of early muscle differentiation effectors, such as MyoD and myogenin, nor the formation of myotubes was affected in cells lacking M19 (data not shown). Moreover, in C2C12 myotubes transiently expressing a M19-specific shRNA associated with GFP, MHCII was only expressed in small patches along the multinucleated cells (Figure 5D, merge; arrowheads), while in control myotubes, i.e. myotubes not expressing the specific shRNA, MHCII was expressed abundantly along the entire cells (Figure 5D, merge; asterisks). Interestingly, as shown in Figure 5E, expression of other late markers of skeletal muscle differentiation such as α-actinin 2, troponin T and MHCI was also affected in M19-deficient cells grown in differentiation medium for 7 days. Altogether, these results show that M19, probably through its regulation of respiratory chain activities, plays a role on late myogenesis.

### M19 is a permissive regulator of insulin secretion in pancreatic β-cells

**Figure 6:**
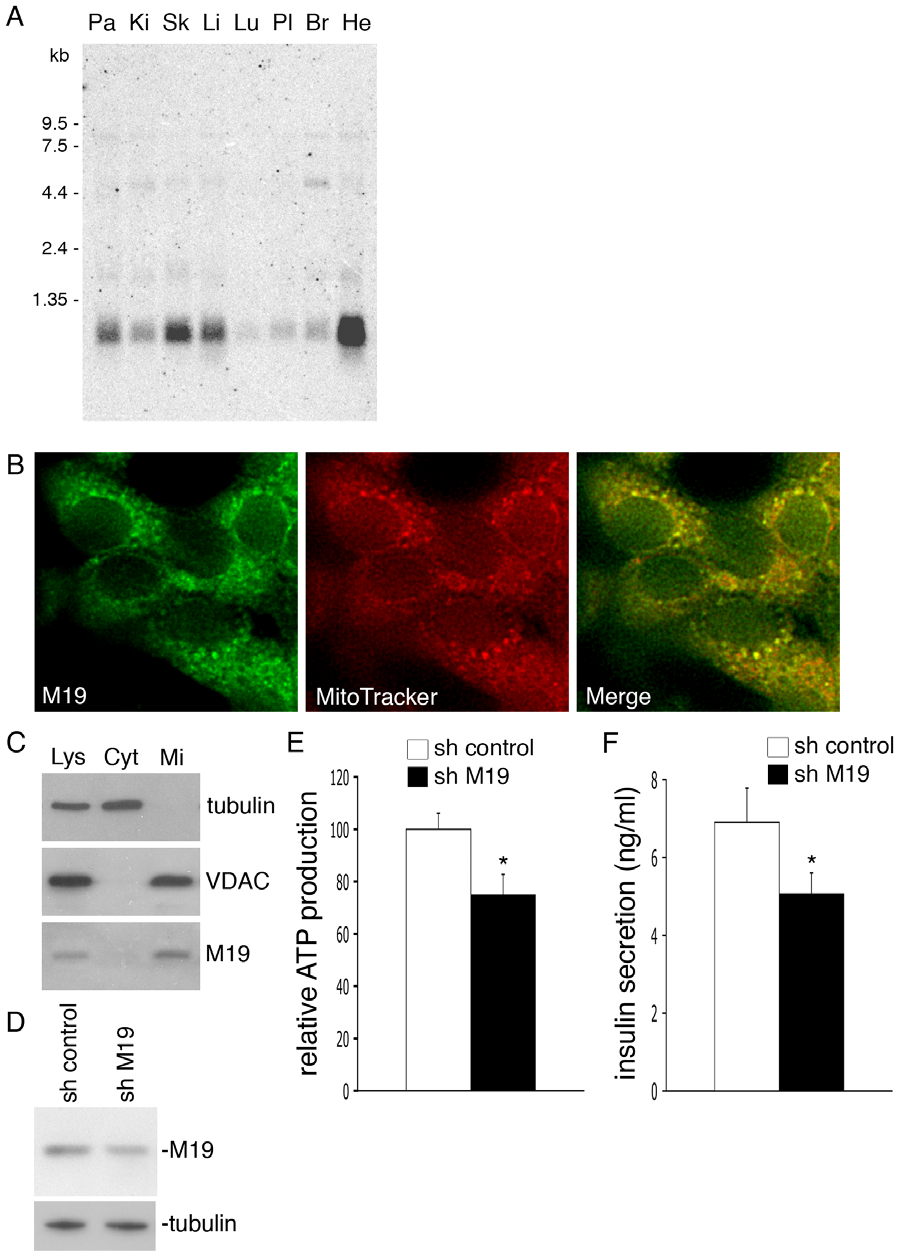
Reduced insulin secretion in M19-deficient INS-1 cells. (**A**) Northern blot analysis of the *M19* gene in human tissues. Pa: pancreas; Ki: kidney; Sk: skeletal muscle; Li: liver; Lu: lung; Pl: placenta; Br: brain; He: heart. Molecular markers are shown on the left. (**B**) Fluorescence microscopy of INS-1 cells double-labeled with the M19-specific P70612 antibody (M19, merge; green) and the MitoTracker dye (MitoTracker, merge; red). (**C**) Cell fractionation of INS-1 cells. Proteins of the total cell lysate (Lys), the cytosolic (Cyt) and the mitochondria (Mi) fractions were subjected to Western immunobloting. The cytosolic protein tubulin, the mitochondrial protein VDAC and M19 are detected. (**D**) INS-1 cells were transfected with a control pHYPER vector (sh control) or with the pHYPER vector encoding a M19-specific shRNA (sh M19). Western immunoblot analysis of the cell extracts shows expression levels of M19 and the control protein, tubulin. ATP production was determined in these cells (**E**), and insulin secretion was measured under basal glucose conditions (**F**). Results are the mean ± SEM of five (**E**), or four (**F**) independent experiments. (*) indicates statistical significance at p<0.05.

A Northern blot performed on human tissue extracts using a specific probe for human M19 mRNA reveals that M19 mRNA is indeed preferentially expressed in striated muscle as previously described [13], but is also especially abundant in pancreas (Figure 6A). It is well established that the mitochondrial ATP/ADP ratio is one of the crucial regulators of insulin secretion in pancreatic β-cells [16]. As we have shown that M19 is a positive regulator of mitochondrial ATP production and therefore can influence the cellular ATP/ADP ratio, we decided to study the involvement of M19 on glucose-induced insulin secretion in pancreatic β-cells. To test this, we used the rat insulinoma cell line INS-1. As shown in Figure 6B, M19 colocalized with the MitoTracker dye into the mitochondria of INS-1 cells. Furthermore, cell fractionation indicated that M19, like the mitochondrial marker VDAC, was mostly present in the mitochondrial extract isolated from INS-1 cells (Figure 6C). To examine whether inhibiting M19 expression has an effect on insulin secretion, we transfected INS-1 cells with a control shRNA vector or with the mouse/rat M19-specific shRNA vector previously used with the mouse C2C12 cell line. After 2 days of culture, M19 expression was reduced by 38% in M19-specific shRNA cells compared to control cells (Figure 6D). Concomitantly, a 25%-decrease in ATP production was observed in M19-deficient INS-1 cells (Figure 6E). Glucose-stimulated insulin secretion was then examined in these cells using increasing amounts of glucose from 2.8 mM (basal) to 8.3 mM (high). Interestingly, a significant 27%-decrease in insulin secretion was observed in M19-deficient INS-1 cells incubated under basal glucose conditions (Figure 6F). However, high-glucose stimulation, or addition of the mitochondrial substrate methyl-succinate, a potent activator of insulin secretion, abolished the reduced insulin secretion observed in M19-deficient INS-1 cells grown under basal glucose conditions (data not shown). Therefore, these experiments suggest that in pancreatic β-cells, M19, through its modulation of ATP production, plays a permissive role on insulin secretion under basal glucose stimulation.

## Discussion

Recently, Sumitani *et al*. published the first characterization of the *C6orf125* gene-encoded protein, M19, in HeLa cells [13]. They showed that M19 is a novel mitochondrial matrix protein associated with the inner membrane of the organelle [13]. Using immunofluorescence microscopy, as well as biochemical assays, we have confirmed the mitochondrial localization of M19 in muscle cells, mostly in the inner membrane. Nevertheless, the lack of hydrophobic transmembrane domain in M19 and the fact that we have also detected the protein in the matrix suggest that M19 might be a matrix protein tightly associated with or anchored to the inner membrane of the organelle. Interestingly, in search for a potential mitochondrial localization signal within this nuclear-encoded protein, we have characterized a N-terminal 13-amino acid long sequence, corresponding to an amphipathic α-helix, which appears necessary and sufficient to target M19 to mitochondria. Furthermore, using RNA interference and over-expression strategies, we have demonstrated that M19 is a positive regulator of mitochondrial oxygen consumption and ATP production. Our results thus suggest that an impaired M19 expression affects the activity of the mitochondrial ATP synthase, highlighting a direct or indirect role for M19 in mitochondrial oxidative phosphorylation.

It has been recently shown that M19 is a component of nucleoids in HeLa cells and, according to its fractionated pattern in nucleoids, it has been suggested that M19 is more precisely associated with the peripheral region of nucleoids [13]. A model for a layered structure of mitochondrial nucleoids has been proposed [14] in which the central core region contains the mtDNA in tight association with proteins involved in replication and transcription, surrounded by a peripheral region containing proteins participating in the translation of mtDNA-encoded proteins and their assembly into respiratory chain complexes. Indeed, mitochondrial ribosomal proteins, chaperone proteins such as LRPPRC which is thought to participate in the assembly of complex IV, and subunits of complex I themselves are found in the peripheral region of nucleoids [14], [17]. Therefore, the fact that M19 is located in this peripheral zone where it would regulate the translation and/or the assembly of respiratory complex proteins is in total accordance with the function of M19 we have described in this manuscript, i.e. the modulation of respiratory chain activities such as oxygen consumption and ATP production.

It is now well established that muscle differentiation is under the control of mitochondrial activity. Indeed, inhibition of mitochondrial protein synthesis by different drugs leads to the inhibition of myoblast differentiation [9], [10]. Here, we have shown that a decreased M19 expression affects late skeletal myogenesis of C2C12 myoblasts, as seen by the reduced expression of various skeletal muscle proteins such as troponin T, α-actinin 2, MHCI and MHCII. This suggests that the decreased ATP production observed in M19-deficient cells could be responsible for the impaired cell differentiation. Nevertheless, it has been shown that myoblast differentiation impairment, induced by inhibition of mitochondrial protein synthesis, was likely not due to modifications of intracellular ATP levels [9], [10]. Interestingly, it has also been shown that respiratory chain impairment in myoblasts can modulate the expression of several nuclear-encoded proteins such as muscle creatine kinase, troponin I, myosin, calcineurin, and the transcription factors myogenin, JNK-dependent ATF2, NF-κB and c-Myc [9], [10], [18], [19], [20]. This recently described signaling pathway, linking mitochondria to the expression of nuclear genes, has been named mitochondria-to-nucleus retrograde signaling, or mitochondrial stress signaling. Biswas and colleagues [18] have further demonstrated that retrograde signaling was mediated by cytosolic Ca^++^ concentration in C2C12 cells. More precisely, mitochondrial membrane potential, mitochondrial ATP production and mitochondrial Ca^++^ uptake can be affected by genetic or metabolic stresses leading to an elevation of the cytosolic Ca^++^ level and thus resulting in the modulation of various protein expression. The data reported in this paper indicate that impairment of M19 expression affects mitochondrial ATP production. This mitochondrial stress leads to the inhibition of the expression of various skeletal muscle proteins essential for terminal differentiation of C2C12 myotubes. Therefore, in M19-deficient cells, it is conceivable that mitochondrial metabolic stresses could induce retrograde signaling resulting in a modified profile of nuclear gene expression and thus in an impaired differentiation.

It is well documented that in pancreatic β-cells, the ATP/ADP ratio is a crucial factor for the coupling of glucose metabolism to insulin secretion [16], and that the determinants of mitochondrial ATP production, such as cytosolic alkalization, glycolysis, mitochondrial oxydative metabolism with also reactive oxygen species production or mitochondrial uncoupling, play a crucial role in this physiological process [11]. Therefore, impairment of mitochondrial oxidative phosphorylation induced by a reduction of M19 expression was likely to induce a defect of insulin secretion in pancreatic INS-1 cells. Nevertheless, the effects we have seen on insulin secretion were only observed under basal glucose conditions. This suggests that M19 is a fine regulator of mitochondrial ATP production and that a rise in mitochondrial substrates, such as glucose or methyl-succinate, is able to bypass the effect of a moderate decrease of M19 expression on insulin secretion.

Type 1 diabetes results from an autoimmune destruction of pancreatic β-cells and various association studies have revealed that multiple genes contribute to disease susceptibility [21]. The major genetic influences for type 1 diabetes have been mapped on human chromosome 6 in a locus containing *MHC class II* genes [22] and the *ITPR3* gene [23], but other genes in or near this locus have been suspected to contribute to type 1 diabetes risk. Interestingly, the *C6orf125/M19* gene is the gene directly centromeric to the *ITPR3* gene on human chromosome 6, these two genes being separated by only 1,000 bp. Knowing that type 1 diabetes is a complex multigenic disorder, it would therefore be of interest to study a possible association of the *C6orf125/M19* gene with this pathology.

In conclusion, our results show that the mitochondrial nucleoid protein, M19, is involved in the regulation of mitochondrial ATP production and therefore is able to modulate various cell activities such as skeletal muscle differentiation/regeneration, or insulin secretion by pancreatic β-cells. This is therefore the first demonstration of the involvement of a nucleoid protein in such physiological processes. Our efforts are now focused on the precise mechanisms through which M19 regulates mitochondrial respiratory chain activity.

## Materials and Methods

### Cells

C2C12 and HeLa cells (ATCC# CRL-1772 and CCL-2, respectively) were routinely cultured in proliferation medium: Dulbecco's Modified Eagle's Medium (DMEM) (Lonza, Switzerland), supplemented with 10% fetal bovine serum (FBS) (Gibco, Invitrogen, Carlsbad, CA), 4.5 g/l glucose, 100 U/ml penicillin, 100 µg/ml streptomycin, 1 mM sodium pyruvate and 2 mM L-glutamine (BioWhittaker, Walkersville, MD). C2C12 cell differentiation was induced by switching FBS to 2% horse serum (Biochrom-Seromed, France) in the medium. The insulin-secreting cell line INS-1 was cultured as previously described [24]. Cells were incubated in an atmosphere containing 5% CO_2_ at 37°C.

### Antibodies and reagents

The polyclonal P70612 antibody was generated at the CRBM animal facility, from the immunization of a rabbit with the full-length bacterially expressed human M19. Mouse monoclonal anti-α-tubulin, anti-myosin heavy chain II, anti-myosin heavy chain I, anti-troponin T and anti-α-actinin 2 were purchased from Sigma-Aldrich (St Louis, MO), mouse monoclonal anti-cytochrome c from BD PharMingen (Franklin Lakes, NJ), mouse monoclonal anti-Penta-His from Qiagen (Hilden, Germany), rabbit polyclonal anti-COXIV from Cell Signaling technology (Danvers, MA), rabbit polyclonal anti-Tom40 (H-300), rabbit polyclonal anti-MyoD (C-20) and rabbit polyclonal anti-myogenin (M225) from Santa Cruz Biotechnology, Inc (Santa Cruz, CA). The MitoTracker Deep Red 633 dye is from Molecular Probes (Eugene, OR).

### Immunostaining

10 µm-cryosat sections of mouse *Tibialis anterior* muscles were saturated with a PBS-BSA (Bovine Serum Albumin) 1% solution during 15 min at 37°C. They were then labeled with primary antibodies diluted in PBS-BSA 1% during 30 min at 37°C. After washing, sections were probed with Texas Red-conjugated anti-mouse IgG (Molecular Probes) and FITC-conjugated anti-rabbit IgG (Molecular Probes).

C2C12 and HeLa cells, grown on glass coverslips, were fixed in 3.7% formaldehyde in PBS followed by a 5 min permeabilization in 0.1% Triton X-100 in PBS. INS-1 cells were seeded on poly-L-lysine (Sigma-Aldrich) coated Lab-Tek Chamber Slide System (Nunc, Rochester, NY). Live INS-1 cells were stained for 45 minutes with the mitochondrion-selective dye MitoTracker DeepRed 633, according to the manufacturer's protocol. After fixation with 2% paraformaldehyde for 20 min, the cells were permeabilized 5 min in 0.1% Triton X-100 and saturated in 2% BSA. Revelation was performed using various antibodies mentioned above. Images were captured with a MicroMax 1300 CCD camera (charge-coupled-device camera; RS-Princeton Instruments, Trenton, NJ) driven by MetaMorph software (v.4.11; Universal Imaging Corp., Westchester, PA) on a Zeiss Axioimager microscope (Carl Zeiss, Oberkochen, Germany).

### SDS-PAGE and Western immunobloting

Samples were boiled 5 min at 95°C, separated on 15% SDS-polyacrylamide gels and transferred onto nitrocellulose or PVDF membranes. Proteins of interest were revealed by specific antibodies mentioned above, followed by enhanced chemiluminescence. Scanned radiographs were quantified with ImageJ (National Institutes of Health, Bethesda, MD).

### Subcellular fractionation and trypsin digestion

To isolate mitochondria from INS-1 cells and C2C12 myoblasts, cells were pelleted by centrifugation for 5 min at 900 g and then washed with PBS. Cell pellets were resuspended in ice-cold mitochondrial isolation buffer (210 mM mannitol, 70 mM sucrose, 1 mM EDTA, 10 mM Hepes pH7.5 with inhibitor protease cocktail) and were homogenized with a Dounce. Nuclei and unbroken cells were removed by centrifugation for 10 min at 800 g at 4°C. Mitochondria were pelleted by further centrifugation for 15 min at 15,000 g at 4°C. Equal protein amount of cytosolic and mitochondrial fractions were separated by SDS-PAGE.

In order to examine trypsin accessibility, 100 µg of mitochondria prepared as described above were resuspended in mitochondrial isolation buffer and treated with increasing amount of trypsin (0, 40, 80 µg/ml) for 20 min on ice. The reaction was stopped by addition of protease inhibitors. Mitochondria were then pelleted by a 20 min-centrifugation at 15,000 g, resuspended in 2X Laemmli buffer and proteins were then separated by SDS-PAGE.

### Plasmid constructions

The full-length open reading frame (orf), as well as a deletion mutant lacking nucleotides 1 to 36 (Δ1–12 mutant), of mouse *M19* were cloned into the pQE-TriSystem vector (Qiagen, Hilden, Germany), which allows the expression of 8xHis-tagged proteins.

The first 39 nucleotides of the mouse M19 orf corresponding to the 13 N-terminus amino acids of M19, or the full-length orf of mouse *M19*, were PCR-amplified and cloned into the pEGFPN1 and pEGFPC3 vectors (Clontech Laboratories, Mountain View, CA).

### RNA interference strategy

siRNA, directed against human M19 cDNA, were synthesized by GeneCust. To optimize the effect, we used a mix of 3 siRNA targeting the following 3 DNA sequences: (5′- CCA CAG ACA CCU UGG AGG Att -3′), (5′- GGA AAU AGA UAA AGG CAU Gtt -3′), (5′- GGA AGA AAC UGC AGG AGA Att -3′). As a control, a siRNA corresponding to a sequence that targets no known messenger was used: 5′- UUC UCC GAA CGU GUC ACG U -3′. HeLa cells, grown in 24-well plates, were transfected with 4 µg of the siRNA mix or the control siRNA using oligofectamine (Invitrogen, Carlsbad, CA).

Oligonucleotides, targeting the mouse *M19* sequence 5′- GCC TGT CCG TGG AAG AGT A -3′, were annealed and then cloned into the pHYPER vector, a modified pEGFPN1 vector allowing GFP expression combined to shRNA expression. The control shRNA sequence used was identical to the control sequence designed for the siRNA strategy. 4 µg of the plasmid were then transfected into C2C12 grown into 60 mm-dishes using JetPei (Polyplus-transfection, New York, NY), according to the instructions provided by the manufacturer.

### ATP assay

C2C12 cells, grown in 60 mm-dishes, were transfected with 4 µg of either the pQE vectors for over-expression experiments or the pHYPER vectors for RNA interference experiments. HeLa cells grown in 24-well plates were transfected with the siRNA mix or the control siRNA alone. One day after, HeLa cells were transfected a second time with the pEGFP/mouse M19 vector for the rescue experiment. Following transfection, C2C12 cells were grown 2 days in proliferation medium while HeLa cells were grown for 1 day under the same conditions. Cells were then trypsinized and 75,000 cells/well were placed in opaque 96-well plates. INS-1 cells were seeded directly on opaque 96-well plates at a density of 40,000 cells/well, and transfection with pHYPER vectors was performed as described below. The CellTiter-Glo Luminescent Cell Viability Assay (Promega, Madison, WI) was then used to determine intra-cellular ATP concentration, according to the instructions provided by the manufacturer. In some experiments, HeLa cells were treated for 1 hour with 62.5 µM oligomycin before determining ATP concentration.

### Cellular respiration assay

C2C12 cells were washed with PBS, trypsinized and resuspended in DMEM. 2,5 million cells in 2 ml were transferred in two sealed thermostated chambers (37°C) of high resolution oxygraph (oxygraph-2k) (Oroboros Instruments, Innsbruck, Austria). Basal respiration was measured, and then non-phosphorylating/uncoupled respiration was determined in the presence of the ATP synthase inhibitor, oligomycin, at a final concentration of 1.25 µM. Carbonylcyanide-3-chlorophenylhydrazone (CCCP), a chemical uncoupler, was titrated into the cell solution to determine maximal oxidative capacity. Then, complex I was inhibited by rotenone (0.25 µM) and complex III was inhibited by antimycin (4.4 µM). This final value of oxygen consumption was subtracted from all the different measurements for the calculation of specific oxygen consumptions. All the reagents were diluted in EtOH and were purchased from Sigma.

### Northern blot

A human multi-tissue Northern blot (Clontech, Mountain View, CA) containing 2 µg of poly(A)^+^ mRNA from adult tissues was used for hybridization analysis. A DNA fragment was amplified by PCR from the full-length human M19 cDNA. The PCR product was labeled with [α-^33^P]dATP and used as a probe on the filter. The filter was then exposed to a phosphor screen. Actin and ubiquitin cDNAs were used as probes to check the presence of similar levels of RNA in each lane.

### Insulin secretion assay

INS-1 cells were seeded on poly-L-lysine coated 24-well plates at a density of 350,000 cells/well. After 3 days of culture, cells were transfected with the control or the mouse/rat M19-specific pHYPER vector using Lipofectamine Plus Reagent (Invitrogen), according to the manufacturer's protocol. Forty eight hours after transfection, cells were washed and pre-incubated for 1 h at 37°C in KRB buffer (108 mM NaCl, 1.19 mM KH_2_PO_4_, 4.74 mM KCl, 2.54 mM CaCl_2_, 1.19 mM MgSO_4_, 18 mM NaHCO_3_) containing 2 g/l BSA in the absence of glucose. After removal of the medium, the cells were incubated for another hour at 37°C in the same buffer in the presence of increasing amounts of glucose (2.8, 5.6, 8.3 mM) with or without methyl-succinate (Sigma-Aldrich). At the end of the incubation period, the medium was collected and insulin was measured by HTRF insulin assay (Cisbio, Bagnols/Cèze, France).
